# Specific interaction of KIF11 with ZBP1 regulates the transport of β-actin mRNA and cell motility

**DOI:** 10.1242/jcs.161679

**Published:** 2015-03-01

**Authors:** Tingting Song, Yi Zheng, Yarong Wang, Zachary Katz, Xin Liu, Shaoying Chen, Robert H. Singer, Wei Gu

**Affiliations:** 1 Department of Pathophysiology, The Key Immunopathology Laboratory of Guangdong Province, Shantou University Medical College, Shantou, Guangdong Province, 515031, China; 2 Department of Anatomy and Structural Biology, Albert Einstein College of Medicine, Bronx NY 10461, USA

**Keywords:** KIF11, ZBP1, Cell polarity, Cell motility, mRNA transport

## Abstract

ZBP1-modulated localization of β-actin mRNA enables a cell to establish polarity and structural asymmetry. Although the mechanism of β-actin mRNA localization has been well established, the underlying mechanism of how a specific molecular motor contributes to the transport of the ZBP1 (also known as IGF2BP1) complex in non-neuronal cells remains elusive. In this study, we report the isolation and identification of KIF11, a microtubule motor, which physically interacts with ZBP1 and is a component of β-actin messenger ribonucleoprotein particles (mRNPs). We show that KIF11 colocalizes with the β-actin mRNA, and the ability of KIF11 to transport β-actin mRNA is dependent on ZBP1. We characterize the corresponding regions of ZBP1 and KIF11 that mediate the interaction of the two proteins *in vitro* and *in vivo*. Disruption of the *in vivo* interaction of KIF11 with ZBP1 delocalizes β-actin mRNA and affects cell migration. Our study reveals a molecular mechanism by which a particular microtubule motor mediates the transport of an mRNP through direct interaction with an mRNA-binding protein.

## INTRODUCTION

mRNA localization is a fundamental mechanism for spatial and temporal control of gene expression (Martin and Ephrussi, 2009). A systematic investigation in *Drosophila* embryos reveals that >70% of endogenous transcripts are localized and the localization of mRNA corresponds to the localization of their encoded proteins (Lécuyer et al., 2007). In a variety of cell types and species, transport of mRNA to a specific cellular compartment enables localized translation, hence generating asymmetric distribution of proteins that are essential for the establishment and maintenance of cellular polarity and structural asymmetry within the cell (Holt and Bullock, 2009; Mili and Macara, 2009).

Several recent studies in yeast and *Drosophila* have illuminated the roles that molecular motors play in the process of RNA localization. These studies have revealed complex mechanisms in which one motor protein or the coordinated action of a few motor proteins act to direct transport and localization of RNAs to their final destination (Gagnon and Mowry, 2011). Both dynein and kinesin motors have been implicated in RNA localization in *Drosophila* oocytes, whereas a type V myosin motor is required for the transport of *ASH1* mRNA in budding yeast (Long et al., 1997; Brendza et al., 2000; Schnorrer et al., 2000; Cha et al., 2002; Duncan and Warrior, 2002; Januschke et al., 2002; St Johnston, 2005). A general model suggests that to localize RNAs, RNA-binding proteins recognize localization elements of their target mRNAs while directly or indirectly connecting to molecular motors. Yeast *ASH1* and *Drosophila* pair-rule mRNAs have provided valuable evidence for this model, in which the unique interactions between RNA-binding proteins and the motors are necessary in order to assemble an mRNP that is fully competent for transport and localization (Darzacq et al., 2003; St Johnston, 2005).

The localization of β-actin mRNAs to the leading edge of migrating cells and to neuronal growth cones of extending axons is associated with cell polarity, cell invasion and neuronal plasticity (Zhang et al., 1999; Condeelis and Singer, 2005; Lapidus et al., 2007). The localization process relies on a trans-acting RNA-binding protein, ZBP1 (also known as IGF2BP1), which contains a unique combination of two RNA recognition motifs (RRMs) and four hnRNP K homology (KH) domains, and specifically recognizes a cis-acting ‘zipcode’ within the 3′ untranslated region (UTR) of β-actin mRNA (Ross et al., 1997; Farina et al., 2003; Hüttelmaier et al., 2005; Chao et al., 2010). Biochemical characterization of the ZBP1 recognition motif reveals that the ZBP1 KH34 region functions as a single unit to interact with the zipcode of β-actin mRNA (Chao et al., 2010). Knockdown of ZBP1 by small interfering (si)RNA impairs cellular adhesion, motility and invadopodia formation (Vikesaa et al., 2006; Gu et al., 2012; Katz et al., 2012). Orthologs of ZBP1 can be found in human, mouse and *Xenopus* (Vg1 RBP/Vera) (Yaniv and Yisraeli, 2002).

Although the majority of localized RNAs are transported along the microtubule cytoskeleton (Bassell et al., 1998; Wilkie and Davis, 2001; Singer, 2008), transport of the ZBP1–β-actin mRNP seems to rely on both microtubules and/or actin filaments (Fusco et al., 2003; Oleynikov and Singer, 2003). Recently, myosin Va (also known as MYO5A) and KIF5A have been shown to play roles in the dendritic and axonal transport of β-actin mRNA (Ma et al., 2011; Nalavadi et al., 2012), and a Rho-mediated signaling pathway operating through a myosin IIB (also known as MYH10) motor was responsible for the sorting of β-actin mRNA in fibroblasts (Latham et al., 2001). It could be hypothesized therefore that in order to properly transport β-actin mRNA, a specific recognition is required for a microtubule or actin motor with ZBP1 that acts as an adaptor protein to associate with the mRNA cargoes. Here, we report the isolation and identification of a kinesin motor, KIF11, which physically associates with ZBP1 *in vivo* to regulate the transport of β-actin mRNA. We characterized the corresponding regions of ZBP1 and KIF11 through which the two proteins interact. Either inhibition of the motor activity of KIF11 or blocking the *in vivo* interaction of ZBP1 with KIF11 delocalizes β-actin mRNA at the cell leading edge and hence alters the cell migration ability. Our study demonstrates a novel mechanism by which KIF11, through a direct interaction with ZBP1, regulates the transport of β-actin mRNP and leads to cell motility.

## RESULTS

### KIF11 associates with ZBP1 and is a structural component of β-actin mRNP

**Fig. 1.**
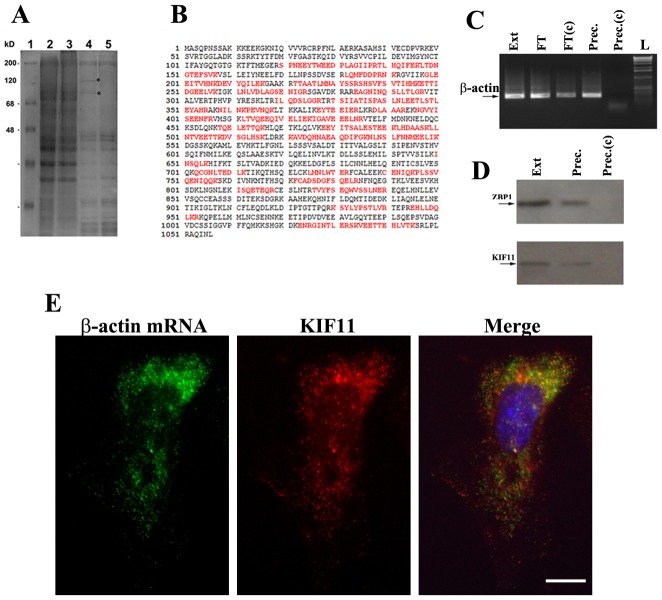
**KIF11 binds to ZBP1 and is a component of the β-actin mRNA complex.** (A) Co-immunoprecipitation was performed to identify the proteins directly associated with ZBP1 in extracts of MDA231/GFP-FLAG-ZBP1 (lane 2) and MDA231/GFP (lane 3) cells. Precipitates were resolved on 4–12% SDS-PAGE and stained with Coomassie Blue. Two protein bands with molecular mass of ∼90 kDa and 110 kDa, were identified in the precipitates of the MDA231/FLAG-GFP-ZBP1 cell extract (lane 4, asterisks), but not in the MDA231/GFP cell extract (lane 5). Western blots showed that the faster migrating band was FLAG-GFP–ZBP1 (not shown). (B) The upper migrating protein band was excised and sent for peptide sequence analysis. Red sequences indicate sequenced peptides that completely match with the sequences of human KIF11. (C) β-actin mRNP was purified from cytoplasmic extracts of MEF cells expressing the β-actin-3′UTR-MBS_24_, using amylose–MBP resin or amylose–MBP-MCP resin. Total RNAs were isolated from starting extracts (Ext), flow-through of amylose–MBP-MCP resin (FT) and amylose–MBP resin [FT(c)], and the precipitates of amylose–MBP-MCP (Prec.) and amylose–MBP resin control [Prec.(c)]. RT-PCR was performed to detect the presence of β-actin mRNA in the RNA samples. Lane L, DNA ladder. (D) Western blots to analyze the presence of KIF11 using rabbit anti-KIF11 antibody and ZBP1 using mouse anti-ZBP1 antibodies in the precipitates. KIF11 and ZBP1 were co-precipitated with β-actin mRNA using amylose–MBP-MCP resin (Prec.), but were not precipitated in the control sample using amylose–MBP resin control [Prec.(c)]. Ext, total extracts. (E) Primary 14-day-old mouse embryo fibroblasts (MEF) of an MBS mouse were cultured on fibronectin-coated coverslips, fixed and processed for immunofluorescence using antibodies against KIF11 (green) and followed by FISH experiments using Cy3-labeled oligonucleotides for β-actin mRNA (red). Blue color indicates the nuclear position. KIF11 was mostly cytoplasmic and colocalized with β-actin mRNA. Scale bar: 10 µm.

In mobile cells, ZBP1 binds to β-actin mRNA and mediates its localized translation. In order to identify potential motor proteins that directly associate with ZBP1 and contribute to the transport process of β-actin mRNA, we prepared extracts from MDA231 breast carcinoma cells in which a FLAG-GFP-tagged human ZBP1 gene was genetically inserted (Gu et al., 2012), treated the cell extracts with RNase I and used anti-FLAG IgG-coated Sepharose to pull down proteins associated with ZBP1. Purified proteins were resolved by 10% SDS-PAGE and visualized by Coomassie Blue staining (Fig. 1A). In contrast to the control precipitation (Fig. 1A, lane 5), two distinct protein bands of ∼90 kDa and 110 kDa (Fig. 1A, lane 4) were isolated. The faster migrating band was identified as FLAG-GFP–ZBP1 (data not shown). Mass spectrometric analysis of the slower migrating band revealed it to be KIF11, a motor protein of the kinesin-5 family (Fig. 1B). To determine whether KIF11 was specifically associated with ZBP1 and β-actin mRNA, we performed another pull-down assay. We prepared primary fibroblasts from embryos of a 14-day-old knock-in mouse strain (MBS strain), in which an MS2 binding site cassette was targeted to the 3′ UTR of the β-actin gene (cells expressing β-actin-MS2 mRNA) (Lionnet et al., 2011). We then used the MS2-binding coat protein (MCP) of the RNA bacteriophage to affinity precipitate the β-actin-MS2 mRNA and its associated proteins. The ability to pull down β-actin-MS2 mRNA by MCP affinity precipitation was assessed by RT-PCR (Fig. 1C). The corresponding precipitates were then evaluated by immunoblotting analysis with anti-KIF11 and anti-ZBP1 antibodies (Fig. 1D). Both ZBP1 and KIF11 were co-precipitated with β-actin-MS2 mRNA (Fig. 1D, Prec.). The specificity of the results was tested in the control experiments in which MCP binding protein was not present [Fig. 1D, Prec.(c)].

### KIF11 colocalizes with β-actin mRNA in fibroblasts

To investigate the subcellular localization of KIF11 and whether KIF11 was colocalized with β-actin mRNA, we isolated and cultured primary fibroblasts from an MBS mouse (MEFs) and performed a combined assay using immunofluorescence analysis with antibodies for KIF11 and *in situ* hybridization with fluorescently labeled MS2 oligonucleotide probes for β-actin mRNA. Distinct β-actin mRNAs were detected at the cell leading edge, owing to the presence of 24 MS2-binding sites in the 3′ UTR of the mRNA (Fig. 1E, left panel). KIF11 was also observed as granules and was distributed in the cytoplasm of the MEF cell (Fig. 1E, middle panel). We superimposed the cell images to show that substantial amounts of KIF11 and β-actin mRNA were colocalized (Fig. 1E, right panel).

### The association of KIF11 with β-actin mRNA depends on ZBP1

**Fig. 2.**
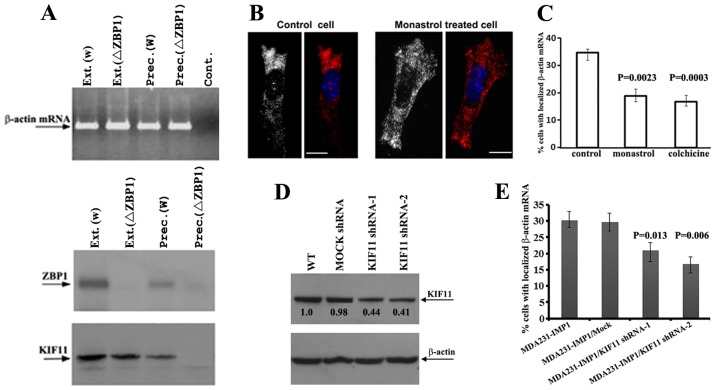
**Inhibition of KIF11 motor function or knockdown of KIF11 expression impairs the localization of β-actin mRNA in MEF cells.** (A) The association of KIF11 with β-actin mRNA is ZBP1 dependent. Cytoplasmic extracts of MEF/MBS cells [Ext (w)] and ZBP1-deleted MEF/MBS cells [Ext (ΔZBP1)] were prepared. β-actin mRNP was purified from the extracts using amylose-resin-attached MBP-MCP [Prec.(w) and Prec.(ΔZBP1)]. Total RNAs were isolated from starting extracts and the precipitates of amylose–MBP-MCP. RT-PCR was performed to detect the presence of β-actin mRNA in the RNA samples. Cont, negative control for RT-PCR. Western blotting was utilized to detect co-precipitated ZBP1(middle panel) or KIF11 (lower panel) with β-actin mRNA. (B) Primary MEF cells were cultured and serum stimulated for 30 min. FISH was performed to determine β-actin mRNA localization in MEF cells treated with monastrol. Blue color indicates the position of nuclei. Scale bars: 10 µm. (C) The localization of β-actin mRNA at the leading edge of the cell was decreased in monastrol- or colchicine-treated cells. A mean of 60 cells was counted from three independent experiments. Data show the mean±s.e.m. Statistical significance was calculated by using Student's *t*-test. (D) Western blots showing the expression levels of KIF11 protein in MDA231-ZBP1 KIF11-shRNA-treated cells. Numbers below the bands reflect the relative levels of KIF11 normalized to β-actin. The arrows indicate the detected proteins. WT, wild type. (E) Graph showing that the percentage of cells with localized β-actin mRNA was significantly reduced in MDA231-ZBP1 KIF11-shRNA-1-treated cells and MDA231-ZBP1 KIF11-shRNA-2-treated cells. The data were derived from three independent experiments. Data show the mean±s.e.m. Statistical significance was calculated by using Student's *t*-test.

We then tested the relationship between KIF11, ZBP1 and β-actin mRNA. We used two stable MEF lines isolated from the MBS mouse strain. In one line, ZBP1 was normally expressed and, in the other cell line, the ZBP1 gene was deleted (Katz et al., 2012). Affinity precipitation of β-actin-MS2 mRNA showed that KIF11 was co-precipitated in the presence of ZBP1, but not co-precipitated when the ZBP1 gene was not expressed (Fig. 2A, lower panel, right two lanes). This suggests that the association of KIF11 with β-actin mRNP was ZBP1 dependent.

### Inhibition of KIF11 activity or knockdown of KIF11 expression impairs the localization of β-actin mRNA

The observation that KIF11 associated with ZBP1 and β-actin mRNA raised the possibility that KIF11 could play a role in the asymmetric transport and localization of the mRNA. To test this assumption, we treated primary fibroblasts with monastrol, a drug that specifically inhibits the motor activity of KIF11 (Mayer et al., 1999), and analyzed the endogenous β-actin mRNA distribution by performing fluorescent *in situ* hybridization (FISH) experiments. Compared with untreated cells, in which ∼34% of the cells showed localized β-actin mRNA at the cell leading edge, localization was decreased to <19% in monastrol-treated cells (Fig. 2B,C). A diminution of β-actin mRNA localization to 17% was also observed in colchicine-treated cells, in which the microtubule structure was destroyed (Fig. 2C). To determine the effect of monastrol treatment on the cytoskeleton, we immunostained the cells with an anti-tubulin antibody and examined levels of β-actin mRNA using RT-PCR, which indicated that both the microtubule structure and the β-actin mRNA expression were unaltered (supplementary material Fig. S1A,B). To further assess the involvement of KIF11 in β-actin mRNA localization, we established two MDA231-ZBP1 stable cell lines in which KIF11 expression was knocked down using the short hairpin (sh)RNA method. Normalized to the internal control of β-actin, KIF11 in shRNA-1-treated cells was downregulated to 44% of its expression in control cells and in shRNA-2-treated cells it was reduced to 41% (Fig. 2D). Experiments indicated that, in comparison to control cells, downregulation of KIF11 delocalized β-actin mRNA from 29% to 20% or 16%, respectively (Fig. 2E). Thus, either inhibition of KIF11 activity or downregulation of KIF11 expression impaired β-actin mRNA localization. We have previously reported that ZBP1, in addition to localizing β-actin mRNA, also plays roles in localizing Arp-16 and α-actinin mRNAs, which are important for focal adhesion dynamics (Gu et al., 2012). To address whether KIF11 has the same effect on the localization of these two mRNAs, we performed FISH assays in KIF11-knockdown cells. The results showed that localization of both Arp-16 (also known as ARPC5) and α-actinin mRNAs at the cell leading edge was significantly reduced (supplementary material Fig. S2). These results suggest the importance of KIF11 in mRNA transport and localization.

### *In vitro* identification of the ZBP1 domain that binds to KIF11

**Fig. 3.**
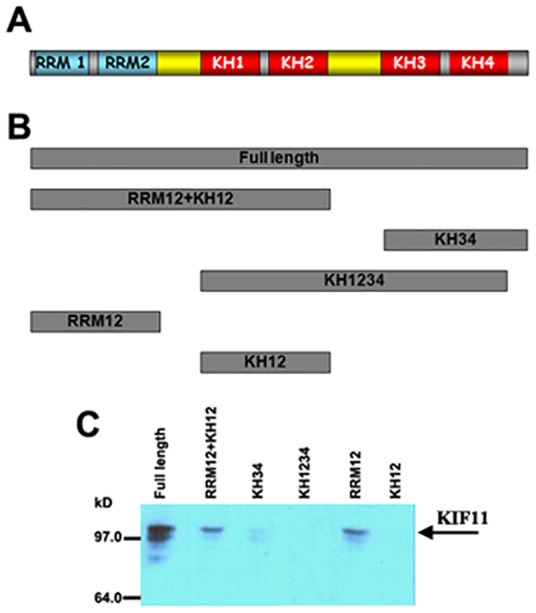
**The RRM12 motif of ZBP1 binds to KIF11 *in vitro*.** (A) A schematic diagram of ZBP1 showing the organization of conserved domains. (B) Representative drawing of recombinant full-length ZBP1, ZBP1-RRM12+KH12, ZBP1-KH34, ZBP1-KH1234, ZBP1-RRM12 and ZBP1-KH12 used in this study. (C) Pulldown assays and western blotting were performed to detect co-precipitated KIF11. The results indicate that the full-length ZBP1, ZBP1-RRM12+KH12 and ZBP1-RRM12 fragments co-precipitated with KIF11 in the cell extracts.

ZBP1 is an RNA-binding protein that contains six canonical domains – two RNA recognition motifs (RRM1 and RRM2, together referred to as RRM12) followed by four hnRNP K homology (KH1–4) domains (Fig. 3A). Previous reports indicate that the region of ZBP1 containing KH3 and KH4 (termed the KH34 domain) binds to the zipcode of β-actin mRNA (Chao et al., 2010). In order to determine the putative region of ZBP1 that was required for interaction with KIF11, we expressed, as maltose-binding fusion proteins (MBP), the full-length ZBP1 and ZBP1 truncation mutants that contained ZBP1-RRM12, ZBP1-RRM12+KH12, ZBP1-KH1234, ZBP1-KH12 and ZBP1-KH34 domains in *Escherichia coli*. (Fig. 3B), and affinity purified the fusion proteins with amylose resin (supplementary material Fig. S3A). We then attached equal amounts of fusion proteins to amylose resin and tested the ability of the truncated ZBP1 protein(s) to pull down endogenous KIF11 from a cellular extract (Fig. 3C). The experiments showed a strong interaction between the full-length ZBP1 and KIF11. Truncated ZBP1 proteins that contained individual KH12, KH34 or KH1234 regions did not bind to KIF11. However, the truncated ZBP1-RRM12 and ZBP1-RRM12+KH12 were co-precipitated with KIF11 (Fig. 3C), demonstrating that the RRM12 motifs of ZBP1 specifically recognized KIF11.

### The tail domain of KIF11 binds to ZBP1 *in vitro*

**Fig. 4.**
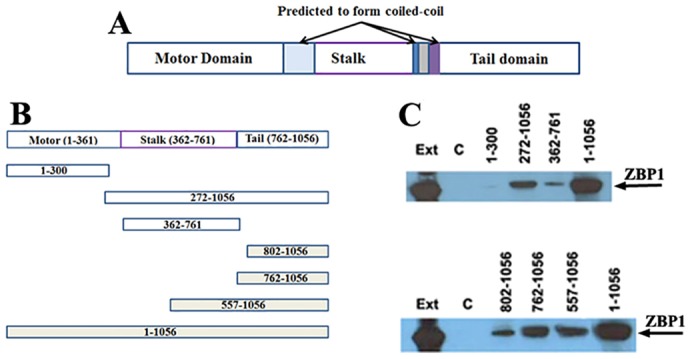
**Identification of the ZBP1-interacting domain of KIF11.** (A) A schematic diagram of KIF11 showing the domain organization. The predicted regions that form coiled-coil structures are indicated. (B) A representative drawing of recombinant fragments of KIF11 used in the experiments to identify the regions responsible for binding to ZBP1. (C) MBP–KIF11 fusion proteins were attached to amylose beads and incubated with extracts of 293T cells. Co-precipitation and western blotting for ZBP1 indicates that the head domain of KIF11 does not bind to ZBP1 and the stalk domain shows a weak interaction with ZBP1, whereas the tail domain (762–1056) fragments were effectively bound to ZBP1. Ext, cell extracts; C, control co-precipitation experiment with MBP-coupled amylose beads.

KIF11 is composed of an N-terminal head domain (motor, residues 1–360), a stalk domain (residues 361–761) and a C-terminal tail domain (residues 762–1056) (Fig. 4A). In addition, three coiled-coil regions within KIF11 were predicted by PAIRCOIL (Berger et al., 1995). In order to identify the potential regions of KIF11 that recognize and interact with ZBP1, we dissected KIF11 protein structure, generated various truncated KIF11 cDNAs by PCR and subcloned the PCR fragments into the plasmid pMalc to express MBP–KIF11 fusion proteins (Fig. 4B). After identifying the purified fusion proteins by SDS-PAGE (supplementary material Fig. S3B), we used equal amounts of proteins for *in vitro* pulldown analyses. We first tested whether the head and stalk domain interact with ZBP1 *in vitro* (Fig. 4C, upper panel). Using extracts prepared from 293T cells, we found that endogenous ZBP1 was efficiently pulled down with the full-length KIF11 and the KIF11 truncation mutant (272–1056) containing both stalk and tail domains. However, the KIF11 stalk domain (362–761) alone showed a relatively weak binding to ZBP1, and no binding was seen with the head domain (residues 1–300). These data suggested that regions other than head and stalk domains interacted with ZBP1. We then focused on the C-terminal region of KIF11 to assess the potential of the tail domain to interact with ZBP1 (Fig. 4C, lower panel). Consistent with the former experiments, full-length KIF11 has the strongest ability to pull down ZBP1 in a cell extract. Two tail-containing truncation mutants of KIF11 with amino acid residues 762–1056 and 557–1056 were able to effectively pull down ZBP1, whereas the 762–1056 tail fragment lacking the 40 amino acid residues at the N-terminus (802–1056) displayed a significantly decreased binding affinity for ZBP1. These results indicate that the tail domain of KIF11 (762–1056) is sufficient and necessary to interact with ZBP1.

### The RRM12 domain of ZBP1 directly interacts with the tail domain of KIF11

**Fig. 5.**
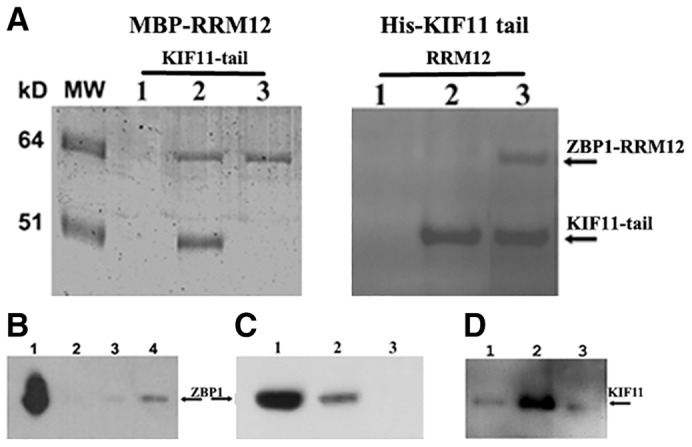
**Direct interaction of the ZBP1 RRM12 motif with the KIF11 tail domain.** (A) ZBP1-RRM12 directly interacts with the KIF11 tail domain (762–1056). Left panel: lane 1, maltose beads without attached proteins were incubated with His-tagged KIF11 tail. Lanes 2 and 3, MBP-RRM12 was attached to maltose beads and incubated with the tail domain of KIF11 or with buffer containing BSA, respectively. Right panel: lane 1, Ni-beads without attached proteins were incubated with ZBP1-RRM12. Lanes 2 and 3, Ni-beads coupled to the His-tagged KIF11 tail fragment were incubated with buffer containing BSA or the ZBP1-RRM12 motif, respectively. Staining after SDS-PAGE was performed with Coomassie Blue. (B) The KIF11 tail domain interacted with endogenous ZBP1. Pull-down experiments were performed with extracts of 293T cells that were infected with lentivirus expressing MBP fusion truncation mutants of KIF11 (1–300) (lane 2), KIF11 (1–363) (lane 3) or the KIF11 tail domain (762–1056) (lane 4) using amylose affinity chromatography. The precipitates were analyzed by western blotting using ZBP1 antibodies. Lane 1, total cell extract of 293T cells. (C) The KIF5A tail domain was not bound to ZBP1 *in vivo*. Pulldown experiments were performed with the extracts of 293T cells infected with lentivirus expressing MBP fusion truncation mutants of the KIF11 tail domain (762–1056) (lane 2) or KIF5A tail domain (806–1032) (lane 3), and analyzed by western blots using ZBP1 antibodies. Lane 1, total cell extract of 293T cells. (D) Analysis of the *in vivo* interaction of the RRM12 domain of ZBP1 with endogenous KIF11. 293T cells were infected with lentivirus expressing His-tagged ZBP1-RRM12 (lane 2) or ZBP1-KH12 (lane 3). Pulldown assays were performed from the extracts of infected cells using Ni-Sepharose beads. The precipitates were analyzed by western blotting using antibodies against KIF11. Lane 1, control pulldown assay with extracts of uninfected 293T cells.

Based on the above results, we anticipated that the recognition of KIF11 by ZBP1 could be through the direct interaction of the ZBP1-RRM12 domain with the KIF11 tail domain. To test this, we coupled MBP-tagged ZBP1-RRM12 to amylose beads, incubated the beads with purified recombinant KIF11 tail fragment and performed co-precipitation experiments. These experiments allowed for detection of the ability of the proteins of interest to form stable complexes. As expected, the tail fragment of KIF11 was able to efficiently bind to immobilized ZBP1-RRM12 to form complexes (Fig. 5A, left panel). We next performed reciprocal experiments in which His-tagged KIF11 tail fragment was coupled to Ni-Sepharose beads and mixed with the recombinant ZBP1-RRM12 fragment. Consistently, the His-tagged KIF11 tail fragment formed complexes with ZBP1-RRM12 (Fig. 5A, right panel). These results indicate the mutual and direct recognition of the tail domain of KIF11 by the RRM12 domain of ZBP1 *in vitro*.

### The KIF11 tail and the RRM12 domain of ZBP1 interact *in vivo*

To determine the *in vivo* interaction of the tail fragment of KIF11 with ZBP1, and the RRM12 domain of ZBP1 with KIF11, we infected 293T cells with lentivirus expressing MBP-fused truncation mutants of KIF11 or His-tagged ZBP1 fragments, respectively. Meanwhile, we also infected cells with a lentivirus expressing the tail domain of KIF5A, which has been shown to play a role in β-actin mRNA localization in neuronal cells (Ma et al., 2011). After analyzing the expression of the fusion protein in infected cells (supplementary material Fig. S4A,B), we used amylose beads or Ni-Sepharose beads for co-precipitation experiments to analyze their *in vivo* mutual interactions. We observed that the tail region (Fig. 5B, lane 4), but not the 1–300 and the 1–361 head regions of KIF11 (Fig. 5B, lanes 2 and 3), was able to co-precipitate with endogenous ZBP1. In contrast to the tail domain of KIF11 (Fig. 5C, lane 2), the KIF5A tail domain had no ability to co-precipitate with endogenous ZBP1 (Fig. 5C, lane 3). We also detected that the RRM12 domain was able to complex with endogenous KIF11 (Fig. 5D, lane 2), whereas the KH12 domain of ZBP1 did not precipitate KIF11 (Fig. 5D, lane 3). These data are consistent with the *in vitro* binding results, suggesting that the *in vivo* physical association of ZBP1 with KIF11 occurs through their corresponding domains.

### Affecting the *in vivo* interaction of ZBP1–KIF11 delocalizes β-actin mRNA

**Fig. 6.**
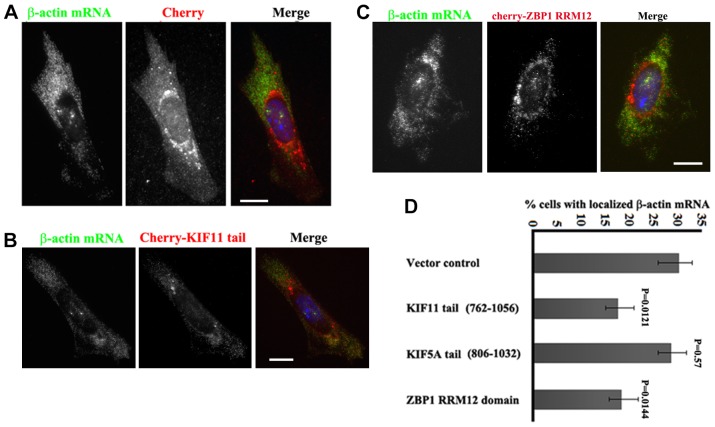
**Impairing the *in vivo* interaction of ZBP1 with KIF11 delocalizes β-actin mRNA from the cell leading edge.** MEF cells were infected with an mCherry lentivirus construct and the constructs expressing the mCherry–KIF11 (702–1056) tail domain, mCherry–KIF5A (806–1032) tail domain or the mCherry–RRM12 motif of ZBP1. After fixation and permeabilization, FISH experiments were performed to detect the localization of β-actin mRNA. (A–C) Representative images showing β-actin mRNA localization in cells expressing mCherry, mCherry–KIF11-tail or mCherry–ZBP1-RRM12. Scale bars: 10 µm. (D) Bars show the percentage of infected cells counted with localized β-actin mRNA at the leading edge. A mean of 50 cells were counted blind per coverslip in three experiments each. Data show the mean±s.e.m. Statistical significance was calculated by using Student's *t*-test.

We postulated that the *in vivo* competition between the KIF11 tail and endogenous KIF11 for binding to endogenous ZBP1 should interfere with β-actin mRNA localization. To determine this, primary fibroblasts isolated from an MBS mouse were separately infected with lentivirus expressing mCherry, or Flag-tagged mCherry fused to the tail (762–1056) of KIF11, the KIF5A tail or ZBP1-RRM12. Infected fibroblasts could be visualized by the mCherry signal and the expression of the protein was analyzed by western blotting (supplementary material Fig. S4C). The localization of β-actin mRNA was visualized by fluorescence *in situ* hybridization. In contrast to the control cells expressing mCherry (Fig. 6A), the localization of β-actin mRNA to the cell leading edge was affected in the cells that expressed the mCherry–KIF11-tail or mCherry–ZBP1-RRM12 fusion proteins (Fig. 6B,C). The percentage of localized β-actin mRNA was decreased from ∼30% to ∼17% in cells expressing the KIF11 tail (762–1056) or to ∼18% in cells expressing the ZBP1 RRM12 domain, whereas the cells expressing the mCherry–KIF5A tail (806–1032) fusion protein showed little effect on β-actin mRNA localization (Fig. 6D). We then analyzed the pattern of cytoplasmic distribution of β-actin mRNAs in infected cells, which was determined as a polarization index using a custom MATLAB script (Gu et al., 2012). Bar graphs (supplementary material Fig. S4D) show that the polarization index of β-actin mRNA in KIF11-tail- or ZBP1-RRM12-expressing cells is lower than that in control and KIF5A-tail-expressing cells, indicating that cells expressing the KIF11 tail or ZBP1-RRM12 show less-polarized distribution of the mRNAs. These data suggest that the dominant-negative expression of the KIF11 tail or the ZBP1-RRM12 fragment impairs the *in vivo* interaction of endogenous ZBP1 and KIF11, resulting in decreased β-actin mRNA localization.

### Blocking the *in vivo* interaction of ZBP1 with KIF11 alters fibroblast motility and increases the invasiveness of breast carcinoma cells

**Fig. 7.**
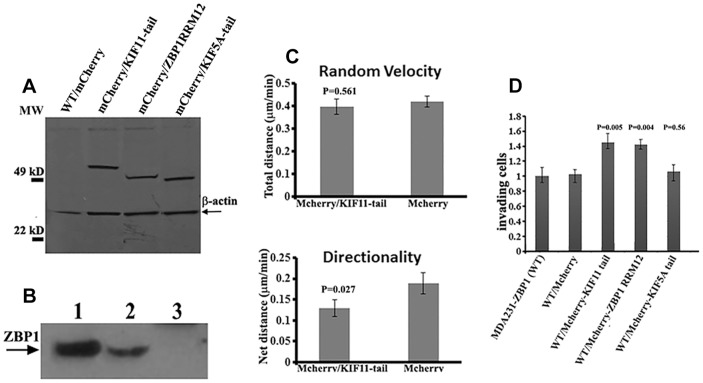
**Expression of the dominant-negative KIF11 tail fragment affects carcinoma cell motility and increases cell invasive ability.** (A) Western blotting was performed to detect protein expression in the stable cells expressing mCherry (not Flag-tagged) (lane 1), Flag-tagged mCherry–KIF11-tail fusion protein (lane 2), Flag-tagged mCherry–ZBP1-RRM12 fusion protein (lane 3) and Flag-tagged mCherry–KIF5A-tail fusion protein (lane 4) using anti-Flag antibodies. The arrow indicates the position of β-actin. WT, wild-type. (B) Pulldown experiments were performed with extracts of MDA231-ZBP1 cells infected with lentivirus expressing Flag-tagged mCherry–KIF11-tail (lane 2) or mCherry–KIF5A-tail (lane 3), using anti-Flag agarose beads. The precipitates were analyzed by western blotting using ZBP1 antibodies. Lane 1, total cell extract of MDA231-ZBP1 cells. (C) Motility analysis was performed by tracking stable cells expressing mCherry or the mCherry-fused KIF11 tail domain under regular growth conditions at 2-min intervals over a 4-h period. Expression of the KIF11 tail domain did not change the random velocity (*P* = 0.0561; two-tailed Student's *t*-test), but decreased the directionality in cell motility (cells expressing mCherry versus cells expressing mCherry–KIF11-tail, *n* = 31 and 32; *P* = 0.027; two-tailed Student's *t*-test). Data show the mean±s.e.m. (D) Overexpression of the KIF11 tail or ZBP1-RRM12 increases the invasive ability of carcinoma cells. Stable cells expressing different mCherry fusion proteins were seeded in serum-free medium into the upper chamber of 8-µm pore Matrigel-coated transwell filters. The lower chamber contained medium with 10% serum. Cells that had invaded to the underside of the filter were stained and counted 16 h later. Invasion was quantified by visual counting of the total cells on the underside of the filter. The relative numbers of invading cells from each assay are normalized to that of the parental MDA231-ZBP1 clone and are represented as a fold change relative to the MDA231-ZBP1. Data shown in the figure represent the mean±s.e.m. of data from three experiments.

To determine the effect of the KIF11–ZBP1 interaction on cell migration ability, we used MDA231/ZBP1 as parental cells to generate stable cell lines expressing mCherry or mCherry fusion proteins of the KIF11 tail, ZBP1-RRM12 or the KIF5A tail. Western blots were utilized to evaluate the expression of the mCherry fusion proteins (Fig. 7A), and a pull-down assay was used to confirm the ability of the KIF11 tail fusion protein to interact with endogenous ZBP1 *in vitro* (Fig. 7B). We then selected two stable cell lines, one cell line expressing mCherry and the other expressing the mCherry–KIF11-tail fusion protein, to test cell motility by using live-cell imaging. This provided an assessment of the differences in motility between genetically identical cell populations that differed only in their level of KIF11 tail expression. The experiments showed that although the random velocity in two cell lines was not substantially changed (Fig. 7C, upper panel), expression of the KIF11 tail led to decreased directionality in cell motility (Fig. 7C, lower panel). These results suggest that dominant-negative expression of the KIF11 tail could affect directional migration of these cancer cells by impairing the endogenous KIF11–ZBP1 interaction.

Gain of ZBP1 function in ZBP1 non-expressing MDA231 cells increases the localization of β-actin mRNA and decreases cell invasion (Gu et al., 2012). To determine whether the *in vivo* interaction of ZBP1–KIF11 might also influence the invasive ability of carcinoma cells, we performed invasion analyses using Matrigel-coated transwells in the presence of 10% FBS. The invasive ability of control MDA231-ZBP1/mCherry cells was not changed compared with that of MDA231 parental cells. However, cells expressing KIF11 tail or ZBP1-RRM12 fusion proteins exhibited nearly a 40% increase in invasion through Matrigel. The observed invasiveness was not seen in the cells that expressed the KIF5A tail (806–1032) (Fig. 7D), suggesting the importance of endogenous communication of ZBP1 and KIF11 in the invasiveness of cancer cells.

## DISCUSSION

KIF11 (also called Eg5 or kinesin-5) is a plus-end kinesin motor with a primary function in the assembly and maintenance of the mitotic spindle (Kashina et al., 1997). The protein has a catalytic motor ATPase domain that mediates its interaction with ATP and microtubules (Valentine and Gilbert, 2007). Loss of KIF11 in mice results in abnormal spindle structure, cell cycle arrest and lethality at embryonic preimplantation (Castillo and Justice, 2007). Overexpression of KIF11 slows neuronal migration and affects the development of leading processes (Falnikar et al., 2011). In fibroblasts, KIF11 has been shown to be involved in microtubule-dependent cell migration in the absence of myosin IIA (Even-Ram et al., 2007). Here, we identify a novel role for KIF11 in the regulation of the transport of β-actin mRNA and cell motility through a direct interaction with ZBP1.

Our studies show that both KIF11 and ZBP1 are components of the β-actin mRNP complex. However, the ability of KIF11 to associate with β-actin mRNP depends on the presence of ZBP1. This provides evidence that ZBP1 is a connector protein for the molecular motor KIF11 and β-actin mRNA cargoes. Previously, it has been shown that the KH34 domain of ZBP1 is required for β-actin mRNA binding (Chao et al., 2008). Here, we reveal that the ZBP1-RRM12 motif is responsible for interacting with KIF11. Moreover, we characterized the tail domain (762–1056) of KIF11 that is necessary for ZBP1 binding. The specificity and the biological significance of the mutual interaction of KIF11 and ZBP1 were further supported by the dominant-negative experiments in which overexpression of the KIF11 tail domain or ZBP1 RRM12 motif markedly delocalized β-actin mRNA and affected cell motility. These results strongly suggest that during the process of RNA localization, ZBP1 enables β-actin mRNP cargoes to be transported along microtubules by simultaneously binding to KIF11 through its RRM12 domain and binding to the zipcode of β-actin mRNA through its KH34 domain.

Previous studies have shown that ZBP1 is able to mediate directional motility of cells and to repress the invasion of breast cancer cells through regulating the localized expression of many adhesion- and motility-related mRNAs, including β-actin, Arp-16 and α-actinin mRNAs (Shestakova et al., 2001; Condeelis and Singer, 2005; Jønson et al., 2007; Gu et al., 2012). These phenotypes have also been observed in our studies presented here, indicating a role for the KIF11–ZBP1 interaction in maintaining directionality in cell motility and suppressing invasiveness of breast carcinoma cells. Based on the findings that KIF11 is responsible for the transport of mRNAs that are important for cell migration and adhesion, and that overexpression of the KIF11 tail or ZBP1-RRM12 truncation mutant affects cell motility and invasiveness, we conclude that the *in vivo* physical interaction of KIF11 with ZBP1 contributes to the transport of mRNAs bound to ZBP1, causing a broader impact on cell polarity and motility.

It is well documented that kinesin motors are required for transporting mRNAs and mRNA-binding proteins in neurons. Hirokawa and colleagues isolated a detergent-resistant RNase-sensitive granule from mouse brain that associated with KIF5, and they showed that transport of RNA-containing granules in dendrites required the C-terminal tail of KIF5 (Kanai et al., 2004). KIF5A was also reported to colocalize with β-actin mRNP in the dendrites of rat cortical neurons (Ma et al., 2011). The involvement of particular kinesin motors in the regulation of the transport dynamics of β-actin mRNA in non-neuronal cells has not been shown previously. Although we identified the requirement of KIF11 for the transport of β-actin mRNP in fibroblasts and carcinoma cells, KIF5A was not shown to be involved in this cellular process. This is not surprising because KIF5A is specifically a neuronal kinesin motor, and it suggests that neuronal and non-neuronal cells could use different mechanisms to transport β-actin mRNA.

Localization of *oskar* RNA in *Drosophila* oocytes not only requires kinesin-1 motor activity, but also needs myosin-V for short-range transport and to mediate the tight anchoring of the mRNA at the posterior pole (Brendza et al., 2000; Krauss et al., 2009). In neuronal cells, both the actin-based motor myosin Va and/or the microtubule-based motor KIF5A were required for the dynamic transport of β-actin mRNA granules (Ma et al., 2011; Nalavadi et al., 2012). In chicken fibroblasts, myosin IIB played a role in localizing β-actin mRNA regulated by a signal transduction pathway (Latham et al., 2001). β-actin mRNA was delocalized in the fibroblasts of myosin-Va-null mice (Salerno et al., 2008). Our findings provide the first evidence that the microtubule motor KIF11 is required for transporting ZBP1 cargoes to the leading edge of the cell. Combining these data, it is most likely that ZBP1 acts as an adaptor protein for different motor machineries to direct RNA localization. The next challenge lies in determining how the microtubule motor is coordinated with the myosin motor to transport and localize ZBP1–β-actin mRNA cargoes.

In summary, our study provides a novel molecular mechanism of how, in mammalian cells, a particular microtubule motor contributes to mRNP transport through the recognition of a specific RNA-binding protein. These data begin to elucidate a sequence of events involved in β-actin mRNA transport: (1) The RNA-binding protein ZBP1 binds to the zipcode of β-actin mRNA through its C-terminal KH34 domain (Farina et al., 2003; Chao et al., 2010), (2) other complementary proteins or translational factors assemble with ZBP1–β-actin mRNA to form a fully functional mRNP complex (locasome) (Hüttelmaier et al., 2005; Pan et al., 2007), (3) the locasome is recognized by a motor through the interaction of KIF11 with the N-terminal RRM12 domain of ZBP1 to form a transport complex or granule, (4) KIF11 transports the locasome along microtubules to the programmed destination and (5) the mRNA is released from the locasome for local translation (Buxbaum et al., 2014).

## MATERIALS AND METHODS

### Isolation and identification of ZBP1-associated proteins

Cultured MDA231/GFP and MDA231/FLAG-GFP-ZBP1 (MDA231 stable cell line constitutively expressing the FLAG-tagged GFP–ZBP1 fusion protein) cells were lysed in ice-cold lysis buffer [10 mM HEPES pH 7.8, 100 mM NaCl, 10 mM KCl, 0.5% NP-40, with a protease inhibitor mixture (Roche)]. After centrifugation at 1200 ***g*** for 5 min, cell debris was removed and supernatants were subjected to an additional step of high-speed centrifugation (16,000 ***g*** for 30 min at 4°C). After adding RNase A (Roche) to a final concentration of 1 µg/ml, the supernatants were incubated with agarose beads immobilized with FLAG-specific monoclonal antibody M2 (Sigma) with gentle rotation for 6 h at 4°C. The beads coupled to ZBP1 and its associated proteins were extensively washed in lysis buffer, eluted and separated on a 10% SDS-polyacrylamide gel. Protein bands, after Coomassie Blue staining, were excised from the gel and subjected to MALDI-TOF at Rockefeller University.

### Isolation of β-actin mRNA complexes

We used a knock-in transgenic mouse line in which a cassette of 24 MS2 binding sites (MBS) was targeted to the 3′ UTR of the β-actin gene (Lionnet et al., 2011). β-actin mRNA complexes were purified from cytoplasmic extracts of embryonic fibroblasts of the knock-in mice using amylose-resin-attached MBP-MCP (MBS binding protein) or amylose-resin-attached MBP, respectively. An aliquot of precipitates was used for extraction of total RNAs for RT-PCR, and the remainder was used for SDS-PAGE and western blotting using antibodies against ZBP1 (Santa Cruz Biotechnology, sc-166344) and KIF11 (Novus Biologicals, NB100-78467).

### Primary cell preparation, fluorescence *in situ* hybridization, immunofluorescence and microscopy

Primary 14-day-old mouse embryo fibroblasts (MEFs) were isolated from an MBS mouse. The MBS cassette provides a means for high-sensitivity fluorescence *in situ* hybridization (FISH), allowing detection and localization of β-actin mRNA molecules in mouse cells (Lionnet et al., 2011). Coverslips were coated with fibronectin (Sigma; working solution, 10 µg/ml; stock solution, 1 mg/ml) for 30 min and washed with PBS. Approximately 2×10^4^ cells were seeded (12-well plates) on the fibronectin-coated coverslips. For serum starvation, MEF cells were washed in Hank's balanced saline solution and incubated overnight in Dulbecco's modified Eagle's medium (DMEM) with 0.5% BSA. Cells were serum stimulated with fresh DMEM plus 10% FBS for 30 min, followed by fixation in 4% formaldehyde. In some experiments, cells were treated with 10% FBS culture medium containing monastrol (working solution, 100 µM; stock solution, 10 mM in DMSO) or with 10% FBS culture medium containing colchicine (working solution, 10 µg/ml; stock solution, 10 mg/ml in ethanol) for 30 min and fixed in 4% formaldehyde. Coverslips containing treated cells were then processed for FISH and immunofluorescence assays as described previously (Lionnet et al., 2011; Gu et al., 2012). For immunofluorescence-FISH combination assays, cells were first incubated with primary and secondary antibodies, fixed in 4% formaldehyde for 5 min and then hybridized with Cy3-labeled oligonucleotides. β-actin mRNA signal was visualized using an Olympus BX61 microscope with a UPlanApo 1003, 1.35 NA objective (Olympus) coupled to an X-Cite 120 PC metal halide light source (EXFO Life Science). Images were captured using MATLAB software. β-actin mRNA was judged to be localized when most of the colored *in situ* signal was asymmetrically distributed at the leading edge (Kislauskis et al., 1997).

### Analysis of cytoplasmic β-actin mRNA distribution

The intracellular polarization and localization of β-actin mRNA was quantified using a custom MATLAB code (Gu et al., 2012). Briefly, the cells were segmented from the FISH images, and the centroid of the nucleus and the intensity-weighted centroid of the cytoplasmic RNA were located. To avoid potential effects that might result from cell shape and size, the polarization index was defined as the distance between the two centroids divided by the radius of gyration of the cell. The radius of gyration was calculated as the root mean square distance of the pixels within the cell area from the centroid of the cell. A higher polarization index indicates a more asymmetric distribution of β-actin mRNA within the cells.

### Cell transfection and infection

For transfection assays, truncation mutants of KIF11 and ZBP1 constructs were transfected, using FuGENE 6 (Roche) or Lipofectamine 2000 (Invitrogen), into MEFs or MDA231 cells that were seeded onto coverslips. After transfection, the MEFs were incubated at 37°C in Dulbecco's modified Eagle's medium (DMEM) supplemented with 10% fetal bovine serum (FBS) for 24 h prior to washing with PBS and fixation using 4% formaldehyde in PBS/5 mM MgCl_2_ for 20 min. For cell infection assays, lentiviral vectors were constructed by subcloning cDNAs encoding mCherry-fused KIF11 or ZBP1 truncation mutants into the pUBC vector. A pGIPZ lentiviral vector encoding an shRNA for KIF11 was purchased from Open Biosystems. Lentiviruses were generated by co-transfecting 20 µg of lentiviral vector and 1 µg of five individual packaging vectors (coding for Gag, Pol, Tat, Rev and VSVG) into HEK 293T cells by using Fugene 6 Transfection Reagent (Roche). Supernatants were collected three times for every 24 h after transfection and filtered through a 0.4-µm membrane. Viral particles were pelleted by centrifugation at 4000 ***g*** using a Lenti-X concentrator (Clontech). The pellets were resuspended in 200 µl of serum-free medium. A volume of 50 µl of the viral suspension was mixed with 0.5 ml of culture medium (serum-free) and used to infect MDA231, MEF or T47D cells that were seeded in a six-well dish at 30% confluence in the presence of 3 µg/ml polybrene (Sigma). Stably infected cell clones were selected by puromycin or sorted by flow cytometry at Einstein College of Medicine.

### Recombinant protein expression and purification

cDNA fragments coding for KIF11 and ZBP1 truncation mutants were PCR amplified and were subcloned into a pMalc plasmid (New England Biolabs) after the MBP gene or in some cases, into a pET21 vector (Novagen). Recombinant protein expressed from pET21 vectors contained a His_6_ tag at the C-terminus. The constructs were transformed into the *Rosseta2 E. coli* strain (New England Biolabs), and expression of the recombinant proteins was induced with 0.5 mM IPTG for 4.5 h. Purification of recombinant proteins was performed according to the manufacturer's protocol with slight modifications. Briefly, the cells were harvested by centrifugation and were resuspended in lysis buffer (50 mM Tris-HCl, 1.5 M NaCl, 1 mM EDTA pH 7.5) supplemented with protease inhibitors. After sonication and clearing of the cell lysate at 14,000 ***g***, the cell extracts that contained MBP fusion proteins were incubated with amylose beads on ice overnight with gentle stirring. After the beads were extensively washed with lysis buffer, the affinity-attached MBP-fusion proteins were eluted with a gradient of maltose ranging from 0.5 to 10 mM. The purity of the proteins was determined by SDS-PAGE and Coomassie Blue staining. Fractions containing pure proteins were pooled and extensively dialyzed against lysis buffer to remove maltose residues.

### *In vitro* pulldown experiments

Cell extracts were prepared from cultured 293T or MDA231-IMP1 cells. Pulldown experiments were performed using MBP–KIF11 or MBP–ZBP1 fusion proteins. Briefly, equal amounts of recombinant MBP fusion proteins (0.1–0.5 µg) were attached to 50 µl of amylose beads and washed twice with 200 µl of lysis buffer. Beads were incubated with 200 µl of cell extract on ice for 4 h with gentle agitation. After centrifugation, the amylose beads were washed five times with 200 µl of lysis buffer, followed by a final wash with 50 µl of lysis buffer. Bound proteins were eluted with 30 µl of 20 mM maltose. Eluted fractions were analyzed by SDS-PAGE and western blotting using monoclonal anti-ZBP1 (kindly provided by Dr Stefan Huttelmaier; Martin Luther University, Halle, Germany) or anti-KIF11 antibodies (Sigma).

### *In vitro* detection of direct protein–protein interactions

His-tagged KIF11 truncation mutants or MBP–ZBP1 fusion proteins were expressed in *E. coli* and purified. About 5 µg of His-tagged tail domain of KIF11 was attached into Ni-beads (Qiagen) and separately incubated with 5 µg of purified MBP1–ZBP1-RRM12 for 6 h at 4°C with continuous stirring. Reciprocally, 5 µg of MBP–RRM12 was attached to maltose beads and incubated with 5 µg of purified tail domain of KIF11 for 6 h at 4°C with continuous stirring. The beads were then pelleted and washed five times with binding buffer. Bound proteins were eluted from Ni-beads with 400 mM imidazole or eluted from maltose beads with 20 mM maltose. Eluted proteins were analyzed by SDS-PAGE and Coomassie Blue staining.

### Cell motility assays

Cells were cultured in wells of a glass-bottomed 24-well plate (MatTek) in DMEM medium containing 10% FBS. Before imaging, the medium was changed to L-15 (Gibco) containing 10% FBS. Cell motility imaging was performed on an IX71 inverted Olympus microscope at 20× using multifield acquisition driven by MetaMorph software. Environmental conditions were controlled for the entirety of the 4-h motility experiment through the use of a heated chamber. Motility analysis was performed on images at 2-min intervals over the 4-h period as a way of fully analyzing a path of cell movement. Tracking was performed in ImageJ using the Manual Tracking and Chemotaxis Tool plug-ins freely available on the ImageJ website. A detailed explanation of motility parameters has been given previously (Soil, 1995). Centroid coordinates were user determined and later used to calculate the motility statistics presented in the results.

### Cell invasion assays

Cell invasion assays were performed using transwells (BD Biosciences) that were pre-coated with Matrigel. Briefly, serum-starved cells were suspended in 400 µl of DMEM supplemented with 0.5% BSA and placed into the transwell chambers (2×10^4^ cells). The chambers were inserted into 24-well culture dishes containing 500 µl of DMEM with 10% FBS. Cells were allowed to invade through the Matrigel for 16 h. The invasive cells underneath the chamber were fixed in 3.7% formaldehyde in PBS for 15 min and stained with 0.2% Crystal Violet in 2% ethanol for 10 min. Noninvasive cells were scraped from the top chambers. The level of invasion was quantified by visual counting of the cells on the underside of the membrane. Each experiment was performed three times, and the results are expressed as means±s.e.m.

## Supplementary Material

Supplementary Material
