# Supplementary material for: Specific interaction of KIF11 with ZBP1 regulates the transport of β-actin mRNA and cell motility
Source: J Cell Sci. 2015 Mar 1;128(5):1001–10. doi: 10.1242/jcs.161679 (PMC4342582; doi:10.1242/jcs.161679)
Supplement: Supplementary Material [file supp_128_5_1001__index.html]

Specific interaction of KIF11 with ZBP1 regulates the transport of β-actin mRNA and cell motility — Supplementary Material 

# Specific interaction of KIF11 with ZBP1 regulates the transport of β-actin mRNA and cell motility

## JCS161679 Supplementary Material

**Files in this Data Supplement:**

- **Supplementary Material**
