# Supplementary material for: Specific interaction of KIF11 with ZBP1 regulates the transport of β-actin mRNA and cell motility
Source: J Cell Sci. 2015 Mar 1;128(5):1001–10. doi: 10.1242/jcs.161679 (PMC4342582; doi:10.1242/jcs.161679)
Supplement: Supplementary Material [file supp_128.5.1001_JCS161679.pdf]

Tingting Song_Suppl Fig.1

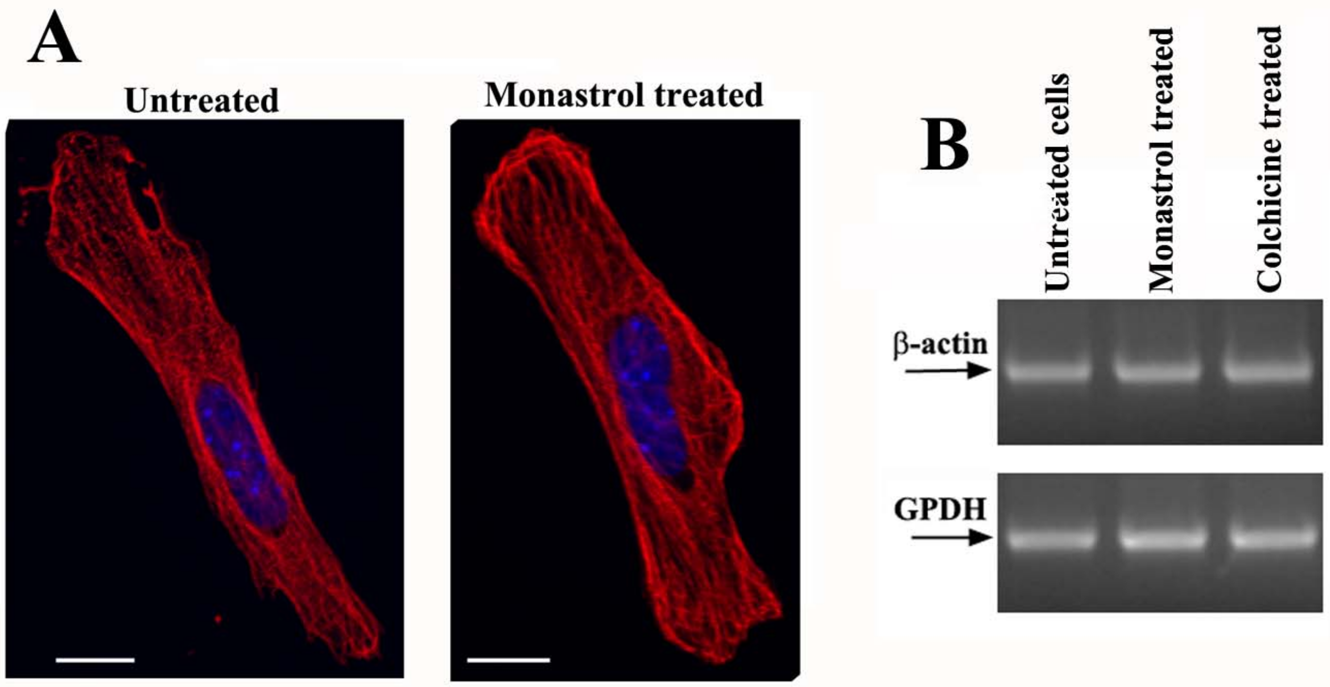

Tingting Song_Suppl Fig.2

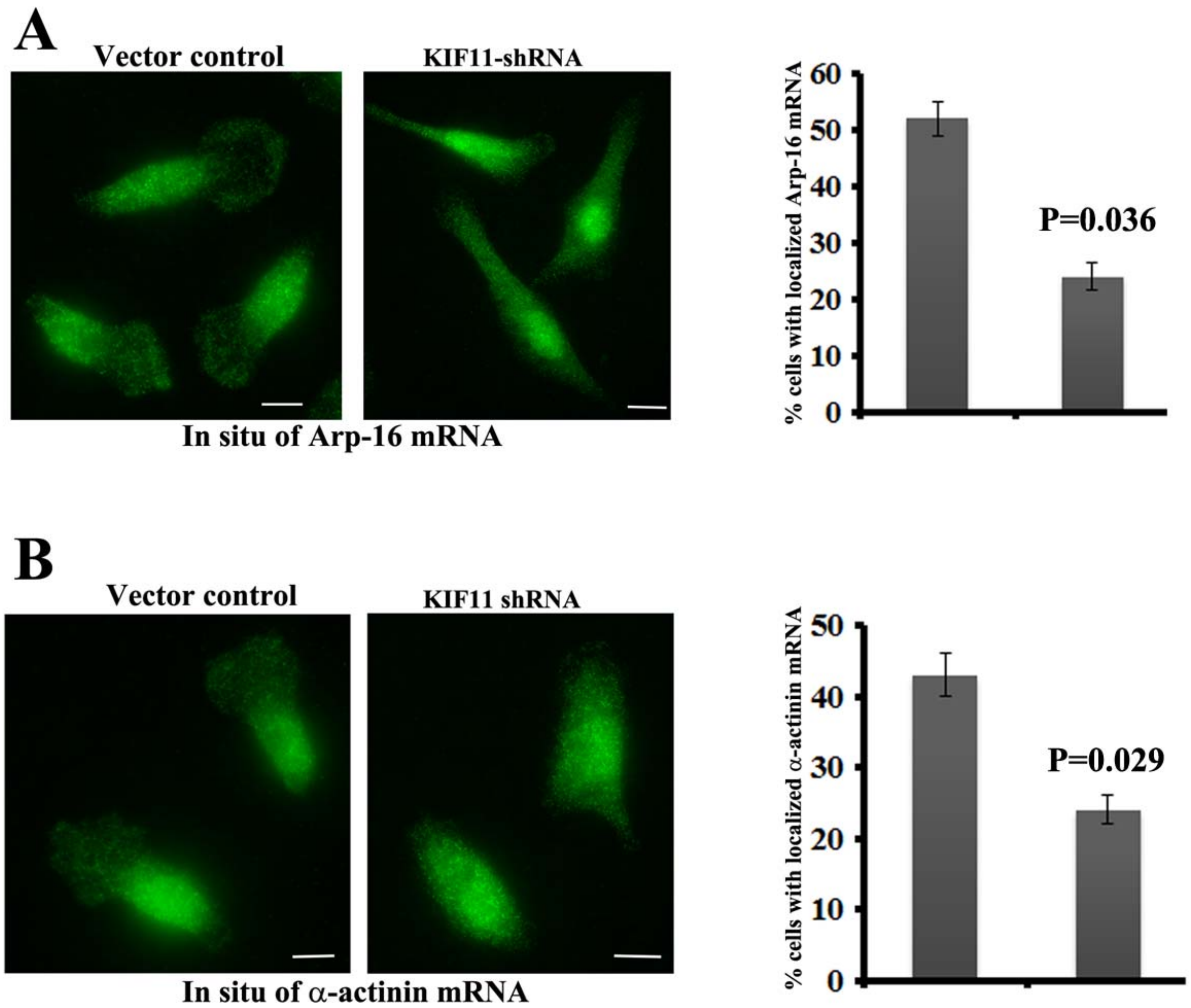

Tingting Song_Suppl Fig.3

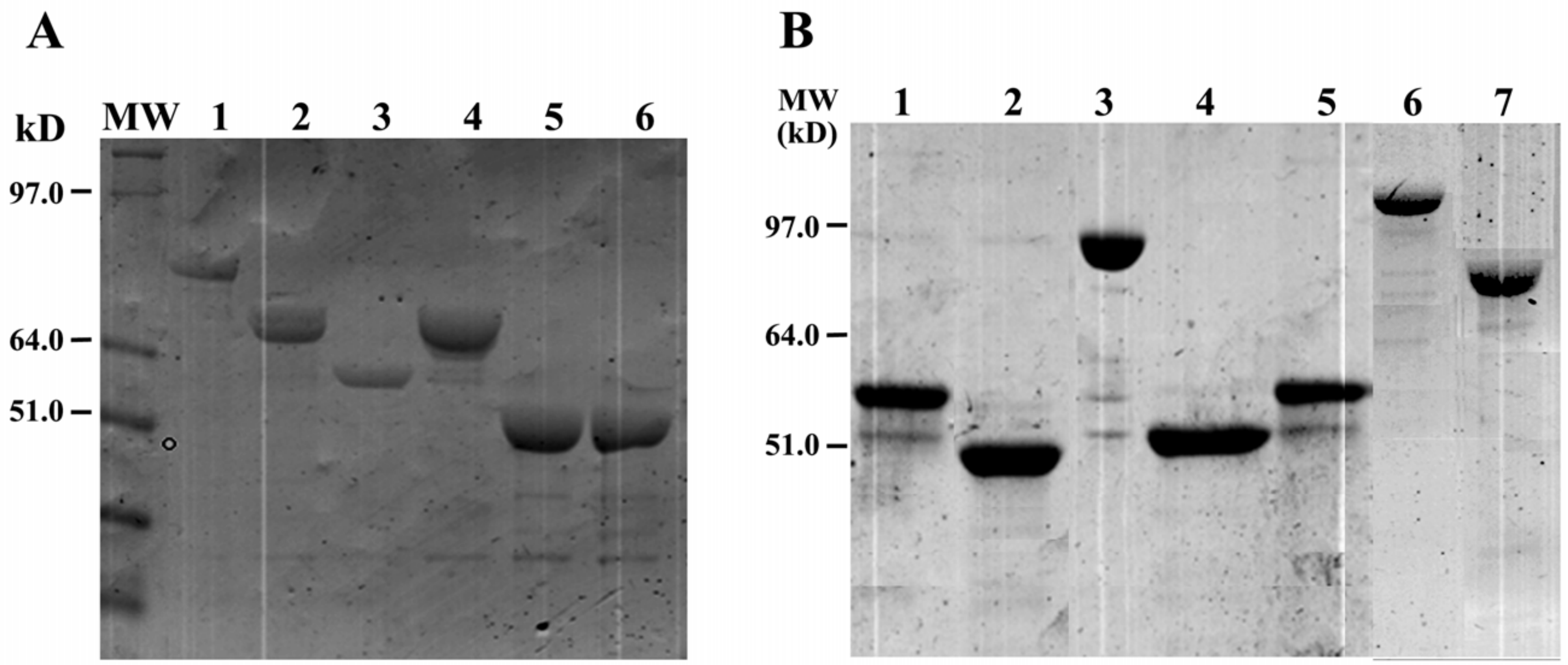

Tingting Song_Suppl Fig.4

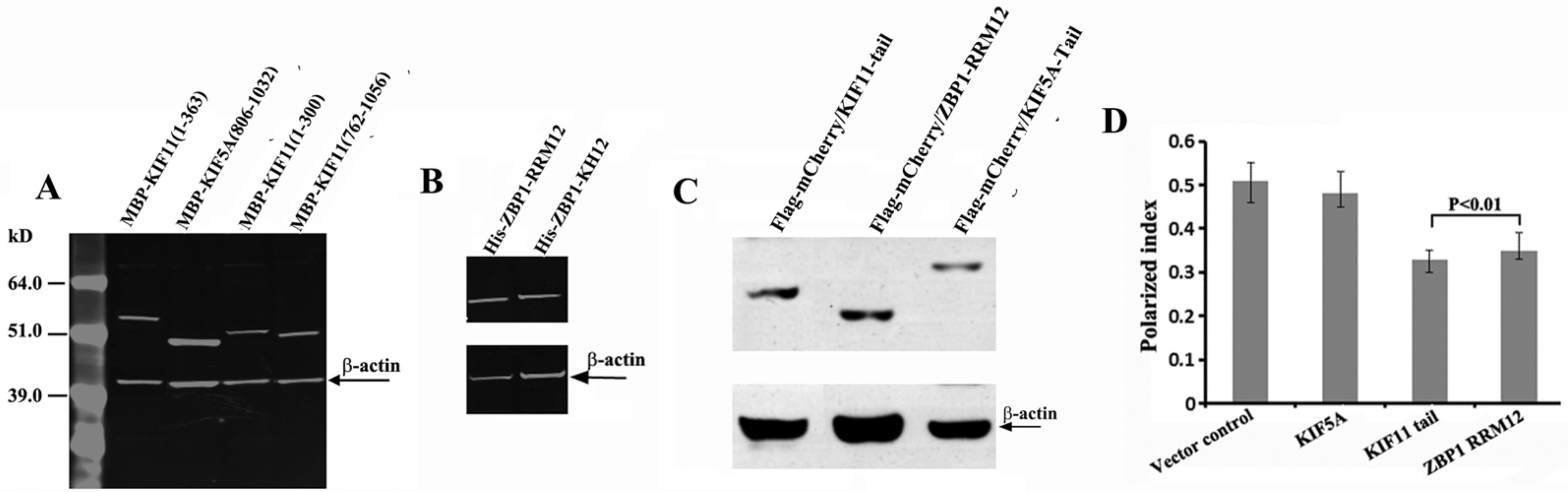

Legends:

Supplemental Figure 1: (A) Examination of changes in microtubule structure in cells with or without monastrol treatment. Embryo fibroblasts of an MBS mouse were cultured on fibronectin-coated coverslips. The cells were treated with monastrol for 30 min, fixed and processed for immunostaining using antibodies against β-tubulin (red). Blue color indicates the cell nuclei after DAPI staining. Scale bar: 10μm. (B) Total RNA was isolated from mouse embryo fibroblasts that were culture and treated with monastrol/colchicine for 30 min. Levels of β-actin mRNA were detected by RT-PCR experiments. Glycerol phosphate dehydrogenase (GPDH) mRNA was used as an internal control.

Supplemental Figure 2: Florescent in situ hybridization (FISH) was performed to detect the localization of Arp-16 mRNA (A) and α-actinin mRNA (B) in MDA231-ZBP1 cells and MDA231-ZBP1/KIF11 shRNA cells. Representive images for RNA localization were shown in the left panels. Right panels indicate the graphs of percentag¬e of cells with localized Arp-16 mRNA (P=0.036) and α-actinin mRNA (P=0.029). Average 100 cells from each cell clone were counted in two independent experiments.

Supplemental Figure 3: MBP-fusion proteins were expressed in E. Coli and were affinity-purified using amylose beads (NEB). (A) Aliquots of purified recombinant MBP-ZBP1 fusion proteins were separated in SDS-PAGE following by Coomassie blue staining. Lane 1, MBP-ZBP1(full length); lane 2, MBP-ZBP1(R12K12); lane 3, MBP-ZBP1(KH34); lane 4, MBP-ZBP1(KH1234); lane 5, MBP-ZBP1(RRM12) and lane 6, MBP-ZBP1(KH12). Molecular weight of the proteins is indicated. (B) Aliquots of purified recombinant MBP-KIF11 fusion proteins were separated in SDS-PAGE following by Coomassie blue staining. Lane 1, MBP-KIF11(362-761); lane 2, MBP-KIF11(1-300); lane 3, MBP-KIF11(272-1056); lane 4, MBP-KIF11(802-1056); lane 5, MBP-KIF11(762-1056); lane 6, MBP-KIF11(full length) and lane 7, MBP-KIF11(557-1056). Molecular weight of the proteins is indicated.

Supplemental Figure 4: (A) Western blots were performed to detect protein expression in lentivirus infected 293T cells expressing MBP-KIF11 head domain (1-363), MBP-KIF5A tail domain (806-1032), MBP-KIF11 head domain (1-300) and MBP-KIF11 tail domain (762-1056) using anti-MBP antibodies. The arrow indicates the position of ß-actin protein. (B) Western blots were used to detect protein expression in letivirus infected 293T cells expressing His-tagged ZBP1-RRM12 and ZBP1-KH12 domain using anti-His-tag antibodies. The arrow indicates the expression of ß-actin protein. (C) Cultured MEF cells were separately infected with lentivirus encoding Flag-tagged mCherry/KIF11-tail, mCherry/ZBP1-RRM12 or mCherry/KIF5A-tail fusions. Expression of the proteins was analyzed by western blots using anti-Flag antibodies. The arrow indicates the expression of ß-actin protein in the samples. (D) ß-actin mRNA localization was quantified by an algorithm that assesses asymmetry (polarization index) using a custom

MATLAB code based on the intensity-weighted centroid of the RNA and nucleus. Polarization index (max=1) was defined as the distance between the two centroids, normalized by the size of the cell (n=30 for each clone; error bars±s.e.m.). A control experiment was performed to address the potential effect of cytoplasmic volume on RNA localization using CellTracker Orange dye (Invitrogen) – no substantial difference between KIF5A-tail and KIF11-tail transfected cells was observed (not shown).
